# Obstetric ultrasound aids prompt referral of gestational trophoblastic disease in marginalized populations on the Thailand–Myanmar border

**DOI:** 10.1080/16549716.2017.1296727

**Published:** 2017-06-02

**Authors:** Kathryn McGregor, Aung Myat Min, Noaeni Karunkonkowit, Suporn Keereechareon, Mary Ellen Tyrosvoutis, Nay Win Tun, Marcus J. Rijken, Gabie Hoogenboom, Machteld Boel, Kesinee Chotivanich, François Nosten, Rose McGready

**Affiliations:** ^a^ Shoklo Malaria Research Unit, Mahidol-Oxford Tropical Medicine Research Unit, Faculty of Tropical Medicine, Mahidol University, Mae Sot, Thailand; ^b^ Julius Centrum Global Health, University Medical Centre Utrecht, Utrecht, The Netherlands; ^c^ Mahidol-Oxford Tropical Medicine Research Unit, Department of Tropical Medicine, Faculty of Tropical Medicine, Mahidol University, Bangkok, Thailand; ^d^ Centre for Tropical Medicine and Global Health, Nuffield Department of Clinical Medicine, University of Oxford, Oxford, UK

**Keywords:** Gestational trophoblastic disease, hydatidiform, molar pregnancy, refugee, ultrasound

## Abstract

**Background**: The use of obstetric ultrasound in the diagnosis of gestational trophoblastic disease (GTD) in high-income settings is well established, leading to prompt management and high survival rates. Evidence from low-income settings suggests ultrasound is essential in identifying complicated pregnancies, but with limited studies reviewing specific conditions including GTD.

**Objective**: The aim of this study is to review the role of ultrasound in diagnosis and management of GTD in a marginalized population on the Thailand–Myanmar border. Antenatal ultrasound became available in this rural setting in 2001 and care for women with GTD has been provided by Thailand public hospitals for 20 years.

**Design:** Retrospective record review.

**Results**: The incidence of GTD was 103 of 57,004 pregnancies in Karen and Burmese women on the Thailand–Myanmar border from 1993–2013. This equates to a rate of 1.8 (95% CI 1.5–2.2) per 1000 or 1 in 553 pregnancies. Of the 102 women with known outcomes, one (1.0%) died of haemorrhage at home. The median number of days between first antenatal clinic attendance and referral to hospital was reduced from 20 (IQR 5–35; range 1–155) to 2 (IQR 2–6; range 1–179) days (*p* = 0.002) after the introduction of ultrasound. The proportion of severe outcomes (death and total abdominal hysterectomy) was 25% (3/12) before ultrasound compared to 8.9% (8/90) with ultrasound (*p* = 0.119). A recurrence rate of 2.5% (2/80) was observed in the assessable population. The presence of malaria parasites in maternal blood was not associated with GTD.

**Conclusions**: The rate of GTD in pregnancy in this population is comparable to rates previously reported within South-East Asia. Referral time for uterine evacuation was significantly shorter for those women who had an ultrasound. Ultrasound is an effective method to improve diagnosis of GTD in low-income settings and an effort to increase availability in marginalized populations is required.

## Background

The epidemiology of gestational trophoblastic disease (GTD) in low-income settings is not well described. Existing studies estimate the rate of GTD to be higher in South-East Asia compared to high-income countries [1], although access to diagnosis and treatment services are likely to be lower [2]. Incidence data vary worldwide, with rates of 1 in 923 pregnancies in the U.S [3] and 1 in 500 pregnancies in South-East Asia [1]. GTD is a spectrum of placental villous trophoblast proliferation from hydatidiform mole to gestational trophoblastic neoplasia, which can progress to invasion and metastasis, increasing the risk of poor maternal outcomes.

There is evidence that GTD risk is associated with low socio-economic status and nutritional deficiencies in prior generations, and that affected women in South-East Asia have a higher rate of malignant disease [4–6]. In high-income settings GTD is often recognized early in pregnancy, initial management (uterine evacuation) occurs without delay, and careful follow-up ensures those with persistent disease are treated successfully, resulting in high survival rates [7]. Conversely, in the challenging environment of a low-resource setting where ultrasound services are scarce, local services for safe uterine evacuation are not available, and particularly when the population is highly mobile and treatment options are limited by the immigration status of the patient, this condition is life threatening and likely to be responsible for maternal mortality. From available data worldwide it is estimated that prior to effective uterine evacuation and chemotherapy, maternal mortality from invasive mole and choriocarcinoma was high at 15% and 100%, respectively [8–10].

Ultrasound is considered a useful tool for identifying hydatidiform mole in pregnancy with studies reporting an overall sensitivity of 44% [11,12], although this is up to 95% for complete hydatidiform moles and as low as 20% for partial hydatidiform moles [11]. Positive predictive values with ultrasound imaging range from 48% to 88% [11,12]. Hydropic abortions have features suggestive of GTD by ultrasound, hence it is not relied upon for definitive confirmation. Pathologic diagnosis can be made following examination of surgical specimens, with immunohistochemical techniques where available [1], although diagnosis during early miscarriage of partial moles can still be difficult due to lack of morphological criteria during the first trimester [13]. Recommended management of GTD pregnancy is surgical, either with suction evacuation and curettage or with hysterectomy if childbearing is completed [1]. As serial serum gonadotrophin (hCG) measurements are used for monitoring, effective contraception post-evacuation avoids confusion in the diagnosis of invasive mole or choriocarcinoma in the following 12 months, and hormonal contraception has not been found to be associated with increased risk of further GTD or to affect hCG normalization [14].

The Shoklo Malaria Research unit (SMRU), based in rural areas of Tak Province on the Thailand–Myanmar border, has been providing antenatal and birthing services in a marginalized refugee and migrant population (mostly of Karen and Burmese origin) for 30 years. The aim of this study was to undertake a retrospective review of case records of GTD cases noting the risk factors for GTD, delays in diagnosis, management and outcomes, particularly comparing outcomes preceding and following the introduction of ultrasound. SMRU has a strong interest in pregnancy malaria, particularly in first-trimester malaria [15], and the relationship between malaria and GTD was also reviewed.

## Methods

### Study site and population

SMRU was established in 1986 in response to the increasing incidence of drug-resistant malaria [16,17], with malaria in pregnancy as one of its priorities due to the high maternal mortality rates from this disease [18]. SMRU integrates health care services with research and has provided free 24-hour healthcare in the refugee camps since 1986 and for the migrant population since 1996. Maela refugee camp, home to SMRU’s longest-standing delivery room, has a current population of 45,000. Maw Ker Thai and Wang Pha clinics serve communities of agricultural migrant workers near the Thailand–Myanmar border. All women are encouraged to attend antenatal clinics as soon as they know they are pregnant. The numbers of women at antenatal clinics have increased yearly, and now over 90% of local pregnant women attend [18]. SMRU birth centres have emphasized training and support of local skilled birth attendants and more than 75% of women who attend SMRU antenatal clinics birth in the facilities [19,20].

Routine obstetric ultrasound became available to SMRU in late 2001 for the refugee population and in 2002 for migrant populations; the quality of the sonographic gestational age estimates has been reported previously [21,22]. Ultrasound machines that have been used over the timeframe of the study include the Toshiba Powervision 7000 machine (Toshiba, Tokyo, Japan), the Fukuda Denshi UF 4100, and the General Electric Voluson i (GE Healthcare, Austria), with 2–5 MHz real-time probes. The turnover of locally trained sonographers has been low and 1–3 have been consistently available at the 3 SMRU clinics, with a total of 13 individual operators during the 20 years. Apart from emergency referrals, images have been reviewed with the sonographers by the site doctor.

A scan at the first antenatal visit and again at 22 weeks is offered to all pregnant women, with complicated pregnancies undergoing more frequent ultrasound monitoring as required. Ultrasound was used as the primary source of dating pregnancies before 24 weeks’ gestation but when this was not reliable (e.g. GTD or blighted ovum) the last menstrual period was used. Symphysis fundal height (SFH) was systematically recorded for women as this was used prior to the introduction of ultrasound. Data on ultrasound and SFH for more than 10,000 pregnancies with normal viable singleton deliveries were used to create local SFH growth charts [23]. Local ultrasound staff are not trained in congenital anomaly scanning. Women with GTD suspected by sonography are counselled and promptly referred to the Thailand public hospital system for care within their facilities. The main reasons for referral are the high risk of severe haemorrhage associated with uterine evacuation [1], and lack of referral networks across to Myanmar.

### Review of pregnancy records

Records of pregnant women who received antenatal care from 1993 to 2013 were reviewed. These records were previously coded for pregnancy outcome as delivered, miscarried (includes GTD) and lost to follow-up (before pregnancy outcome was known). All miscarriage cases were reviewed, GTD being identified separately, and data were extracted from these records including: fundal height measurements [23], the time from first antenatal visit to treatment (recorded in days), actual treatment received, follow-up, and final diagnostic category of GTD where available. Detailed case notes, in particular the histopathological reports from the Thailand Government Hospital system, were not available to SMRU but hospital caseworkers recorded basic data on discharge before women returned to the field sites. Only women with a histopathologically confirmed diagnosis of GTD have been included in this review. Unfortunately, information on the type of molar cases from complete to partial mole was limited. Obviously some women were referred as suspected GTD but did not have positive histopathology results.

### Statistical analysis

Data were analysed using SPSS for Windows™ (Version 20, SPSS Inc.). Continuous normally distributed data were described by their means and compared with the Student’s *t*-test, while non-normally distributed data were described by their median and compared with the Mann–Whitney U test. Confidence intervals (CI) were calculated using the method described by Robert Newcombe [24]. Percentages were calculated for categorical data and compared using the χ^2^ test or Fisher’s exact test. For cross-tabulations Bonferroni correction was used to adjust for pairwise comparisons within each row.

Factors associated with a GTD pregnancy were compared by univariate analysis and odds ratios (OR) were calculated with a 95% CI. To assess independent risk factors associated with GTD a multivariate logistic regression model was fitted using the variables that were significantly associated with GTD from the univariate analysis. A two-sided *p*-value of less than 0.05 was deemed to be statistically significant. Due to collinearity between age and parity, and parity and a history of miscarriage, these were omitted from the final model.

In this setting the risk of malaria is not stable across the duration of pregnancy [15]. It is more likely to be detected earlier in pregnancy and more likely to be detected at the first antenatal screen. For this reason the proportion of women with malaria was confined to a fixed window, before a gestational age of 16 weeks.

## Results

There were 57,004 records available for analysis. Of these women 41,445 (72.7%) delivered, 5347 (9.4%) miscarried including 103 cases of GTD and 10,212 (17.9%) were lost before the outcome of pregnancy was known. The incidence of GTD was 103 of 57,004 pregnancies between 1993 and 2013: 1.8 (95% CI 1.5–2.2) per 1000.

### Demographic characteristics and risk factors associated with GTD

The median (IQR) gestational age at presentation was significantly lower in women with GTD compared to no GTD: 11.3 (8.0–15.6) weeks (n = 99) vs 13.6 (8.8–21.9) weeks (n = 55,524), *p* < 0.001; as were the haematocrit (mean ± standard deviation): 32.8 ± 5.4% (n = 88) and 33.8 ± 4.3% (n = 52,093) respectively, *p* = 0.024; and the body mass index (BMI) (mean ± standard deviation) in women who presented in the first trimester: 19.8 ± 3.0 kg/m^2^ (n = 59) and 21.0 ± 3.0 kg/m^2^ (n = 18,479), *p* = 0.003, respectively. Not all women had a palpable S.F.H or reliable estimate of gestational age. The proportion of first SFH measurements that reached the 90th centile or higher for gestational age between weeks 10 and 16, was significantly higher for women with GTD compared to without GTD: 80.0% (20/25) vs 33.6% (3270/11,077), *p* < 0.001; OR 7.9 (95% C.I 3.0–21.1).

A higher proportion of teenagers and women aged 40 years or more with GTD was noted, but this was only statistically significant for older women (Table 1). As expected in women of older age, higher parity and numbers of miscarriage were identified (Table 2) but these factors were collinear. There was no significant effect of smoking observed. On multivariate analysis being 40 years of age or more and being underweight (first trimester BMI < 18.5 kg/m^2^) were both significantly and independently associated with a raised risk for GTD: adjusted odds ratio (AOR) 2.56 (95% CI 1.28–5.13) and 3.82 (95% CI 2.41–6.06), respectively (Table 2).

**Table 1.** Proportion of GTD by five-year age groups.

Age category in years	GTD (n = 103)	No GTD (n = 56,869)^a^
<15	0^b^ (0.0)	54 (0.1)
15–19	18 (17.5)	9298 (16.3)
20–24	23 (22.3)	15,934 (28.0)
25–29	25 (24.3)	13,323 (23.4)
30–34	15 (14.6)	9634 (16.9)
35–39	11 (10.7)	6237 (11.0)
40–44	8 (7.8)^c^	2144 (3.8)
45–49	3 (2.9)^c^	245 (0.4)

*Notes*: ^a^Age missing for 32 women; ^b^not included in comparison as value is zero; ^c^*p* < 0.05 adjusted for pairwise comparisons within each row using Bonferroni correction.

**Table 2.** Patient demographics in women with GTD and non-GTD pregnancies.

	GTD n (%)	Non-GTD n (%)	Odds ratio univariable (95% CI), *p*-value	Adjusted odd ratio (95% CI), *p*-value
**Age category (years)**				
< 40	92 (89.3)	54,480 (95.8)	Reference	Reference
≥ 40	11 (10.7)	2389 (4.2)	**2.73 (1.46–5.10), *p* = 0.004**	**2.56 (1.28–5.13), *p* = 0.008**
**Parity**				
Parity ≤ 4	84 (81.6)	50,394 (88.7)	Reference	Omitted
Parity > 4	19 (18.4)	6423 (11.3)	**1.78 (1.08–2.92), *p* = 0.029**	
History of miscarriage*				
No miscarriage	42 (56.0)	27,427 (65.4)	Reference	Omitted
At least one miscarriage	33 (44.0)	14,533 (34.6)	1.48 (0.94–2.34), *p* = 0.089	
**Smoker**				
Non-smoker	67 (71.3)	34,844 (72.8)	Reference	Not applicable
Smoker	27 (28.7)	13,047 (27.2)	1.08 (0.69–1.68), *p* = 0.735	
**Underweight BMI < 18.5 kg/m^2^**				
Normal or overweight	38 (64.4)	15,192 (82.2)	Reference	Reference
Underweight	21 (35.6)	3287 (17.8)	**2.55 (1.50–4.36), *p* = 0.001**	**3.82 (2.41–6.06), *p* < 0.001**

*Notes:* Missing data: age n = 32; parity n = 84; smoking n = 9019; BMI in trimester one n = 9980.

*Primigravida cannot have a history of miscarriage and was therefore excluded. OR and AOR values in bold were significant *p*<0.05.

### Diagnosis

Twelve of the 103 GTD cases (11.7%) were diagnosed before ultrasound became routinely available.

Most women, 83.5% (76/91), with ultrasound diagnosis had the classical ‘vesicular pattern’ or ‘grape-like’ appearance at the ultrasound scan at the first antenatal visit. Partial mole was observed in 6.6% (6/91) of women: five by the presence of a fetus on ultrasound (all without a fetal heartbeat) and one from one of the six histological reports available.

There were 21 women who had a pregnancy test done of whom 23.8% (5/21) had a negative test result and one of whom had a serum hCG available and confirmed as > 200,000 IU/L.

### Length of time to referral

Of the 88.3% (91 of 103) of women with an early ultrasound there was a significantly shorter median number of days, 2 (IQR 2–6; range 1–179), to referral into the Thailand public hospital system compared to the period before ultrasound was routinely available, 20 (IQR 5–35; range 1–155) days (n = 11, excludes one maternal death before referral), *p* = 0.002. From the period where ultrasound was available and despite equal availability of ultrasound in both marginalized groups, the median time to referral was longer for migrant women, 3 (IQR 2–13; range 1–179) days, compared to refugee women, 1 (IQR 1–2; range 1–115) day, *p* < 0.001.

The presentation of range, rather than just IQR, demonstrates a high number of days from the date of the first antenatal visit until referral into the Thailand public hospital system despite ultrasound availability.

### Outcome of pregnancy

There were no notes on the outcome for one migrant woman diagnosed with GTD by ultrasound and she has not been included in further analysis. Of the 102 remaining women, one died (1.0%) of haemorrhage at home in a village outside the camp during the time before ultrasound had become available and without receiving any treatment. On review the fundal height was large for gestational age. Mortality in women diagnosed with GTD without an ultrasound scan was 9.3% (95% CI 6.8–10.0) (1/12) and zero amongst the 90 women who were scanned.

Initial treatment for women with documented follow-up (after excluding the woman who died) included 94.1% (95/101) with uterine evacuation and 5.9% (6/101) with total abdominal hysterectomy (TAH). A total of 9.9% (10/101) of women had TAH as a further four women received this operation after local recurrence following uterine evacuation. There was a trend for more women to receive a TAH before ultrasound was available, but this was not significant.

Invasive disease confirmed by histology and/or chest radiograph was diagnosed in 11.9% (12/101) of cases. There were five cases where chemotherapy from the Thailand public hospital was documented in SMRU case records, but this is most likely an underestimate.

Of the 94.1% (95/101) of women with uterine evacuation, 5.3% (5/95) had recurrence of GTD. The follow-up of these patients and systematic recording of hCG levels into the primary record held by SMRU were poor as they were routinely recorded into the patients’ handheld medical record books, which were not available for this review.

### Recurrence in a new pregnancy

For recurrence in a new pregnancy, women with tubal ligation (n = 11) and TAH (n = 10) were removed from the denominator as new pregnancy was prevented, leaving 80 women. After post-uterine evacuation and apparent cure, 2 women (2.5%) were diagnosed as GTD on their subsequent pregnancy: one at 12 months and one at 16 months post-treatment. Both were treated with repeat uterine evacuation without chemotherapy. We were unable to confirm the incidence of recurrent pregnancy without GTD in the remaining 78 women so this figure may underestimate recurrence.

### Contraception

TAH provided definitive contraception in 9.9% (10/101) of women. Contraceptive uptake after initial treatment of GTD was not documented in 18.8% (19/101) of cases. Of the 72 remaining women, 40.3% (29/72) declined contraception, 22.2% (16/72) took the oral contraceptive pill, 15.3% (11/72) had a sterilization (tubal ligation), 11.1% (8/72) opted for condoms, 9.7% (7/72) opted for medroxyprogesterone and one (1.4%; 1/72) had an intra-uterine device placed.

### Malaria and GTD

In women with GTD who had a known gestational age, 76.8% (76/99) had their first antenatal visit before 16 weeks. The proportion of malaria at the first antenatal visit in women less than 16 weeks’ gestational age was compared. In women with GTD compared to without GTD the proportion of *P.falciparum* infection was 1.3% (1/76) vs 2.4% (780/32,173); for *P.vivax* infection 2.6% (2/76) vs 2.5% (815/32,173); and for malaria smear negative 96.1% (73/76) vs 95.0% (30,578/32,173); with no significant difference for all comparisons.

## Discussion

The incidence of GTD in this population, 1.8 (95% CI 1.5–2.2) per 1000, is comparable to previously reported data for South-East Asian women with an estimated rate of 1.7 (1.4–2.1) to 2.3 (1.8–2.9) per 1000 pregnancies depending on the inclusion criteria used [25]. In this cohort one pregnant woman died from complications of GTD. She presented before routine ultrasound scanning was introduced and there have been no deaths since that time from GTD [18].

Routine ultrasound at the first antenatal visit reduced the referral time for definitive treatment with uterine evacuation significantly. Local sonographers have been trained in gestational age assessment and non-classical scans still resulted in diagnostic indecision, leading to rescanning or in some cases measurement of hCG. In this setting, where advanced laboratory facilities are not available on site, quantitative hCG results arrive at field clinics 3–7 days after blood samples are taken. Ultrasound at SMRU was originally introduced to improve the assessment of gestational age which has proved particularly difficult in this area where literacy rates are low [26], and an accurate last menstrual period is difficult to obtain [21,22]. In the absence of access to timely hCG assessment, locally available obstetric ultrasound services in low-resource settings are likely to improve outcomes by giving real-time information about a broad array of pathologies, including GTD [27]. This study adds to evidence from low-income countries that obstetric ultrasound is essential to identify and manage complicated pregnancies, and that provision of training and equipment in these settings should be encouraged [28]. Efforts to improve sonographer recognition of non-classical GTD if machines have the capacity for colour Doppler could also improve earlier diagnosis. This may be especially important in the migrant women who are highly mobile and hence the opportunistic chance to confirm GTD at a single ultrasound scan should be maximized.

Compared to previous observations, similar risk factors associated with GTD have been identified including high maternal age and high parity. The association between GTD and being underweight, which has not previously been described, may reflect the fact that women in this population often reach institutional care after being unwell for a prolonged period of time. Smoking in this population was not significantly associated with the risk of GTD but data may be confounded by the high rate of household smoke exposure due to the use of charcoal for cooking [29]. SFH above the 90th centile can be helpful in the diagnosis of GTD but confirmatory testing with ultrasound or hCG measurement is needed as a next step, both of which can be challenging in a rural environment if ultrasound is not locally available [22]. Measurement of urine hCG may not suffice as this test may be negative when serum hCG is very high, as observed in a limited number of cases in this data-set [30].

Areas of improvement in clinical practice include the need for a greater effort in effective contraception and enhanced communication with subsequent health authorities to obtain detailed discharge information. Low uptake of contraception could result from miscommunication as this marginalized population do not speak Thai, so careful explanation of this condition to the woman in her own language is required. Post-evacuation hCG documentation in this cohort was poor and is a definitive action point for future service provision, particularly with the proportion of 2.0% repeat GTD, which is higher than reported rates elsewhere of 1% [31,32].

As far as we are aware this is the first time the risk of GTD and malaria has been compared and there was limited power to show an association despite systematic screening for malaria in this population [15].

The lack of availability of the histopathological diagnosis of complete or partial mole in this study is the main limitation of this report. There may be case ascertainment bias as monitoring and referrals are likely to have improved since the introduction of ultrasound, beyond the merits of the investigation itself, as education and awareness improve amongst staff and infrastructure and referral systems strengthen over time. In addition there is incomplete documentation of chemotherapy and follow-up for those returning from the Thailand Health System. The low rate of post-evacuation hCG results would underestimate recurrence and mortality. Nevertheless reporting outcomes of GTD in marginalized populations is important to understand their risk, and for programmatic improvement to reduce pregnancy-related maternal mortality.

## Conclusion

In women who had an ultrasound scan offered early in pregnancy there was no maternal mortality and a significantly reduced time to referral for definitive management of GTD. Ultrasound use in marginalized populations and in rural areas should be encouraged.

## Paper context

Evidence linking the use of obstetric ultrasound to reduced maternal mortality in limited-resource settings is lacking. This study demonstrates a significant reduction in referral time for definitive management of molar pregnancy, and a trend for lower mortality and morbidity, following introduction of ultrasound, in a challenging low-resource setting with a highly mobile population. Improving access to obstetric ultrasound in these settings to aid identification and management of complicated pregnancies is likely to improve outcomes.

## References

[1] Lurain JR. Gestational trophoblastic disease I: epidemiology, pathology, clinical presentation and diagnosis of gestational trophoblastic disease, and management of hydatidiform mole. Am J Obstet Gynecol. 2010 12;203:531–7. Elsevier Inc. 20728069 10.1016/j.ajog.2010.06.073

[2] Groen RS, Leow JJ, Sadasivam V, et al Review: indications for ultrasound use in low- and middle-income countries. Trop Med Int Health. 2011 12;16:1525–1535. 21883723 10.1111/j.1365-3156.2011.02868.x

[3] Hayashi K, Bracken MB, Freeman DH, et al Hydatidiform mole in the United States (1970-1977): a statistical and theoretical analysis. Am J Epidemiol. 1982;115:67–77. 7055132 10.1093/oxfordjournals.aje.a113281

[4] Coullin P, Diatta AL, Boufettal H, et al The involvement of the trans-generational effect in the high incidence of the hydatidiform mole in Africa. Placenta. 2015;36:48–51. 25468544 10.1016/j.placenta.2014.10.017

[5] Nizam K, Haider G, Memon N, et al Gestational trophoblastic disease: experience at Nawabshah Hospital. J Ayub Med Coll Abbottabad. 2009;21:94–97. 20364752

[6] Talati NJ The pattern of benign gestational trophoblastic disease in Karachi. J Pak Med Assoc. 1998;48:296–300. 10087749

[7] Savage P Molar pregnancy. Obstet Gynaecol. 2008;10:3–8.

[8] Soper JT Gestational trophoblastic disease. Obstet Gynecol. 2006;108:176–187. 16816073 10.1097/01.AOG.0000224697.31138.a1

[9] Bracken MB Incidence and aetiology of hydatidiform mole: an epidemiological review. Br J Obstet Gynaecol. 1987;94:1123–1135. 3322372 10.1111/j.1471-0528.1987.tb02311.x

[10] Choi M, Lee C, Smith H, et al Epidemiology In: Hancock B, Seckl M, Berkowitz R, editors. Gestational trophoblastic disease [Internet]. 4th ed. 2015 Available from http://isstd.org/gtd-book/chapter3/

[11] Kirk E, Papageorghiou AT, Condous G, et al The accuracy of first trimester ultrasound in the diagnosis of hydatidiform mole. Ultrasound Obstet Gynecol. 2007;29:70–75. 17201012 10.1002/uog.3875

[12] Fowler DJ, Lindsay I, Seckl MJ, et al Routine pre-evacuation ultrasound diagnosis of hydatidiform mole: experience of more than 1000 cases from a regional referral center. Ultrasound Obstet Gynecol. 2006;27:56–60. 16273594 10.1002/uog.2592

[13] Johns J, Greenwold N, Buckley S, et al A prospective study of ultrasound screening for molar pregnancies in missed miscarriages. Ultrasound Obstet Gynecol. 2005;25:493–497. 15818571 10.1002/uog.1888

[14] Braga A, Maestá I, Short D, et al Hormonal contraceptive use before hCG remission does not increase the risk of gestational trophoblastic neoplasia following complete hydatidiform mole: a historical database review. BJOG An Int J Obstet Gynaecol [Internet]. 2016 7;123:1330–1335. 10.1111/1471-0528.13617 26444183

[15] Moore KA, Simpson JA, Paw MK, et al Safety of artemisinins in first trimester of prospectively followed pregnancies: an observational study. Lancet Infect Dis. 2016;16:576–583. 26869377 10.1016/S1473-3099(15)00547-2 PMC4835584

[16] Carrara VI, Lwin KM, Phyo AP, et al Malaria burden and artemisinin resistance in the mobile and migrant population on the Thai-Myanmar border, 1999-2011: an observational study. Plos Med. 2013;10:e1001398. 10.1371/journal.pmed.1001398 PMC3589269 23472056

[17] Carrara VI, Sirilak S, Thonglairuam J, et al Deployment of early diagnosis and mefloquine-artesunate treatment of falciparum malaria in Thailand: the Tak Malaria initiative. Plos Med. 2006;3:e183. 16719547 10.1371/journal.pmed.0030183 PMC1470664

[18] McGready R, Boel M, Rijken MJ, et al Effect of early detection and treatment on malaria related maternal mortality on the north-western border of thailand 1986-2010. Plos One. 2012;7:e40244. 22815732 10.1371/journal.pone.0040244 PMC3399834

[19] Hoogenboom G, Thwin MM, Velink K, et al Quality of intrapartum care by skilled birth attendants in a refugee clinic on the Thai-Myanmar border: a survey using WHO safe motherhood needs assessment. BMC Pregnancy Childbirth [Internet]. 2015;15:17 Available from http://www.scopus.com/inward/record.url?eid=2-s2.0-84924180418&partnerID=tZOtx3y1 10.1186/s12884-015-0444-0 PMC4332741 25652646

[20] Parr M, Dabu CP, Wai NS, et al Clinical audit to enhance safe practice of skilled birth attendants for the fetus with nuchal cord: evidence from a refugee and migrant cohort. BMC Pregnancy Childbirth [Internet]. 2014;14:76 Available from http://www.pubmedcentral.nih.gov/articlerender.fcgi?artid=3943506&tool=pmcentrez&rendertype=abstract 10.1186/1471-2393-14-76 PMC3943506 24552462

[21] Rijken MJ, Gilder ME, Thwin MM, et al Refugee and migrant women’s views of antenatal ultrasound on the thai burmese border: A mixed methods study. Plos One. 2012;7:e34018. 10.1371/journal.pone.0034018 PMC3325974 22514615

[22] Rijken MJ, Lee SJ, Boel ME, et al Obstetric ultrasound scanning by local health workers in a refugee camp on the Thai-Burmese border. Ultrasound Obstet Gynecol. 2009;34:395–403. 19790099 10.1002/uog.7350 PMC3438883

[23] White LJ, Lee SJ, Stepniewska K, et al Estimation of gestational age from fundal height: a solution for resource-poor settings. J R Soc Interface [Internet]. 2012;9:503–510. Available from http://www.pubmedcentral.nih.gov/articlerender.fcgi?artid=3262426&tool=pmcentrez&rendertype=abstract 10.1098/rsif.2011.0376 PMC3262426 21849388

[24] Newcombe RG Two-sided confidence intervals for the single proportion: comparison of seven methods. Stat Med. 1998;17:857–872. 9595616 10.1002/(sici)1097-0258(19980430)17:8<857::aid-sim777>3.0.co;2-e

[25] Atrash HK, Hogue CJ, Grimes DA Epidemiology of hydatidiform mole during early gestation. Am J Obstet Gynecol. 1986;154:906–909. 3963081 10.1016/0002-9378(86)90482-5

[26] Carrara VI, Hogan C, De Pree C, et al Improved pregnancy outcome in refugees and migrants despite low literacy on the Thai-Burmese border: results of three cross-sectional surveys. BMC Pregnancy Childbirth. 2011 1;11:45. 21679475 10.1186/1471-2393-11-45 PMC3142536

[27] Hofmeyr G, Haws RA, Bergstr MS, et al Obstetric care in low-resource settings: what, who, and how to overcome challenges to scale up? Int J Gynaecol Obstetrics. 2009; 107 Suppl 1:S21–44, S44-5. 10.1016/j.ijgo.2009.07.017 19815204

[28] Åhman A, Kidanto HL, Ngarina M, et al “Essential but not always available when needed” - an interview study of physicians’ experiences and views regarding use of obstetric ultrasound in Tanzania. Glob Health Action [Internet]. 2016;9:31062 Available from: http://www.ncbi.nlm.nih.gov/pubmed/27452066 10.3402/gha.v9.31062 PMC4958909 27452066

[29] Turner C, Turner P, Carrara V et al. High rates of pneumonia in children under two years of age in a south east Asian refugee population. Plos One. 2013;8:e54026. 10.1371/journal.pone.0054026 PMC3539989 23320118

[30] Tabas JA, Strehlow M, Isaacs E A false negative pregnancy test in a patient with a hydatidiform molar pregnancy. N Engl J Med [Internet]. 2003;349:2172–2173. Available from: http://www.ncbi.nlm.nih.gov/pubmed/14645652 10.1056/NEJM200311273492221 14645652

[31] Berkowitz RS, Im SS, Bernstein MR, et al Gestational trophoblastic disease. Subsequent pregnancy outcome, including repeat molar pregnancy. J Reprod Med. 1998;43:81–86. 9475154

[32] Sand PK, Lurain JR, Brewer JI Repeat gestational trophoblastic disease. Obs Gynecol. 1984;63:140–144. 6320076

